# Complete Genome Sequence of *Leisingera aquamixtae* R2C4, Isolated from a Self-Regenerating Biocathode Consortium

**DOI:** 10.1128/MRA.00833-19

**Published:** 2019-09-05

**Authors:** Lina Bird, Brian J. Eddie, Anthony P. Malanoski, Princess Pinamang, Sarah M. Glaven

**Affiliations:** a National Research Council, Washington, DC, USA; b Center for Bio/Molecular Science and Engineering, Naval Research Laboratory, Washington, DC, USA; c Department of Biology, Prairie View Agricultural and Mechanical University, Prairie View, Texas, USA; d The Washington Center for Internships and Academic Seminars, Washington, DC, USA; University of Southern California

## Abstract

Here, we present the complete genome sequence of Leisingera aquamixtae R2C4, isolated from the electroautotrophic microbial consortium biocathode MCL (Marinobacter-*Chromatiaceae*-*Labrenzia*). As an isolate of a current-producing system, the genome sequence of L. aquamixtae will yield insights regarding electrode-associated microorganisms and communities. A dark pigment is also observed during cultivation.

## ANNOUNCEMENT

Leisingera aquamixtae R2C4 was isolated from the biocathode MCL (Marinobacter-*Chromatiaceae*-*Labrenzia*), a self-regenerating microbial consortium that uses electrons supplied by the cathode to drive CO_2_ fixation and O_2_ reduction. Biocathode MCL was enriched from Atlantic Ocean seawater at Rutgers University Marine Field Station, Tuckerton, NJ (1), and metagenomic sequencing showed a low-diversity community of *Gammaproteobacteria* and *Alphaproteobacteria* (2). Isolation followed enrichment of biofilm scrapings in an iron sulfide gradient tube (3) and was carried out by streaking the turbid layer from the tube onto an artificial seawater agar plate supplemented with 5 mM sodium acetate. The resulting colonies grew well on half-strength Zobell’s marine agar plates (4). One isolate, designated R2C4 and initially identified as a Phaeobacter sp. (5), produced a dark pigment. Several *Phaeobacter* spp. have since been reclassified as *Leisingera* spp. (6), which led us to identify R2C4 as a *Leisingera* sp.

*Leisingera* spp. are aerobic and moderately halophilic members of the *Rhodobacteraceae* family with the ability to utilize methyl halides as a carbon source (7) and to produce antimicrobial compounds thought to aid in symbiotic relationships (6). There are seven named species of the genus *Leisingera* (6–12), including two (L. caerulea and L. aquimarina) that were isolated from cathodes (13, 14). Analysis of the genome of Leisingera aquaemixtae R2C4 will support our efforts to use multiomics in characterizing biocathodes, help identify potential extracellular electron transfer (EET) proteins and pathways, and determine R2C4’s role in electroactive microbial communities.

Genome sequencing was performed using the Pacific Biosciences RS II sequencing platform (DNA Link USA, Inc., San Diego, CA). Genomic DNA was extracted from 2 ml Difco marine broth using the Wizard genomic DNA purification kit (Promega). One microgram of DNA was used to prepare a 10-kb insert library and sequenced using two single-molecule real-time (SMRT) sequencing cells and P4-C2 chemistry. Default parameters were used for all software, unless otherwise noted. Filtering and preassembly were performed with SMRT Analysis v2.3.0 HGAP.2 (PacBio). This resulted in 17,730 filtered and preassembled sequence reads with a mean length of 7,272 bp, an *N*_50_ value of 8,071 bp, and 75× genome coverage. *De novo* assembly (via SMRTpipe HGAP.2 and SMRTpipe Celera assembler) and consensus polishing (SMRTpipe Quiver) yielded one closed circular genome and nine smaller contigs, of which four are closed circular DNA and five, with low sequence coverage (<30×), are not closed, as summarized in Table 1. Annotation was performed using NCBI’s Prokaryotic Genome Annotation Pipeline. The genome is predicted to contain 4,424,497 bp, 4,093 protein-coding genes, 56 tRNA-encoding genes, and 4 rRNA operons. BLAST alignments of the 16S rRNA genes (locus tags R2C4_03015, R2C4_13205, R2C4_16735, and R2C4_16910) showed 100% identity to Leisingera aquaemixtae SSK6-1 (formerly Phaeobacter aquaemixtae SSK6) (15). Digital DNA-DNA hybridization (16) confirmed that R2C4 belongs to *L. aquaemixtae,* showing 77.2% identity (74.2 to 80% confidence interval [CI]) to *L. aquaemixtae* CECT8399. An NCBI BLAST search of the relevant genes showed that R2C4 contains a predicted indigoidine synthesis operon, *igiRBCDFE*, which shares 96% to 98% nucleotide identity with the operon in *L. aquaemixtae* CECT8399 (11). The dark color of R2C4 colonies suggests that indigoidine, a blue pigment with antimicrobial properties, is produced.

**TABLE 1:** Sequence characteristics of assembled contigs

Accession no.	Length (bp)	GC content (%)	No. of CDS^*a*^	Circularization, coverage (×)
CP041155	13,809	66.8	13	No, low
CP041156	175,590	66.01	167	Yes
CP041157	121,606	58.22	170	Yes
CP041158	59,812	68.7	55	Yes
CP041159	3,820,802	64.52	3,694	Yes
CP041160	6,320	65.9	7	No, <30
CP041161	14,269	68.5	11	No, <30
CP041162	26,129	65.5	21	No, <30
CP041163	32,785	67.8	29	No, <30
CP041164	108,983	65.2	86	Yes

a CDS, coding sequences.

### Data availability.

The complete sequence of *Leisingera aquamixtae* R2C4 is available under NCBI BioProject number PRJNA480465, with the annotated sequences available in GenBank under accession numbers CP041155 to CP041164. Raw sequencing reads are deposited in the Sequence Read Archive under accession number SRR965965.
